# Effects of Virtual Reality-Based Interventions on Pain Catastrophizing in People with Chronic Pain: A Systematic Review and Meta-Analysis

**DOI:** 10.3390/jcm14113782

**Published:** 2025-05-28

**Authors:** Claudio Carvajal-Parodi, Pedro O. Rossel, Alejandra Rodríguez-Alvarado, Francisco Guede-Rojas, Jesús G. Ponce-González

**Affiliations:** 1 Escuela de Kinesiología, Facultad de Odontología y Ciencias de la Rehabilitación, Universidad San Sebastián, Lientur #1457, Concepción 4030000, Chile; 2 Programa de Doctorado en Ciencias de la Actividad Física y del Deporte, Universidad de Cádiz, Campus Puerto Real, Avda. República Saharaui s/n, 11519 Cádiz, Spain; 3 Departamento de Ingeniería Informática, Universidad Católica de la Santísima Concepción, Concepción 4030000, Chile; prossel@ucsc.cl; 4 Centro de Investigación en Biodiversidad y Ambientes Sustentables (CIBAS), Universidad Católica de la Santísima Concepción, Concepción 4030000, Chile; 5 Centro del Dolor, Las Condes, Santiago 7550000, Chile; alejandrarodri2000@yahoo.com; 6 Exercise and Rehabilitation Sciences Institute, School of Physical Therapy, Faculty of Rehabilitation Sciences, Universidad Andres Bello, Santiago 7591538, Chile; francisco.guede@unab.cl; 7 ExPhy Research Group, Department of Physical Education, Instituto de Investigación e Innovación Biomédica de Cádiz (INiBICA), Universidad de Cádiz, 11002 Cádiz, Spain; jesusgustavo.ponce@uca.es

**Keywords:** chronic pain, pain catastrophizing, virtual reality, systematic review, meta analysis

## Abstract

**Background**: Chronic pain affects millions worldwide, influenced by biological, psychological, and social factors. Catastrophizing predicts chronic pain outcomes, increased pain intensity, and worsening recovery. Virtual reality (VR) interventions offer innovative pain management strategies, but their effects on catastrophizing remain unclear. **Methods**: This systematic review and meta-analysis followed the PRISMA guidelines. Studies involving adults with chronic musculoskeletal pain, VR-based interventions, and randomized controlled trials were included. The primary outcome was pain catastrophizing. Searches were conducted in PubMed, CINAHL, Scopus, WoS, and PEDro until May 2025. The risk of bias was assessed using Cochrane RoB-2. Meta-analysis calculated effect sizes using mean differences (MD) and standardized mean differences (SMD) with fixed and randomized-effects models. **Results**: Of 306 records, 244 were screened, 19 underwent full-text review, and two additional studies were identified via Google Scholar. Nine studies were included, eight of which were meta-analyzed. The interventions included eight immersive and one non-immersive VR studies, lasting 3 to 12 weeks. A small but statistically significant effect was found when comparing VR-based interventions with controls (SMD = −0.26 [−0.48; −0.04]). Psycho-cognitive VR-based interventions had a significant effect (SMD = −0.32 [−0.56; −0.09]), while exercise-based VR did not (MD = −0.11 [−4.36; 4.14]). Immersive VR showed a small but significant effect when compared to non-intervention or sham controls (SMD = −0.37 [−0.75; −0.00]). However, when compared to all types of comparators, the effect was not statistically significant (SMD = −0.25 [−0.51 to −0.00]). Heterogeneity was moderate and not significant (*p* > 0.05). **Conclusions**: VR-based interventions, particularly immersive with psycho-cognitive approaches, show potential in reducing pain catastrophizing. Future randomized trials are needed to elucidate VR’s efficacy in managing pain catastrophizing.

## 1. Introduction

Pain is the leading cause of healthcare consultations globally, and its chronic form garnered increasing clinical and epidemiological interest [1]. In the United States, the annual prevalence of chronic pain ranges from 11% to 40%, with persistent pain conditions representing three of the top four causes of years lived with disability. In the United Kingdom, recovery rates remain low, with only 5.4% of individuals experiencing chronic pain achieving significant improvement [2].

The understanding of chronic pain has evolved significantly, particularly following the recent update to the definition of pain by the International Association for the Study of Pain (IASP), which emphasizes the psychosocial dimensions, individual experiences, and the effects of pain on overall well-being [3]. Chronic pain, which persists for at least three months, can be categorized into primary, where no identifiable cause exists, and secondary, which stems from a specific underlying condition [4]. Several factors, including biological, psychological, and social elements, contribute to the transition from acute to chronic pain, positioning it as a clinical challenge that demands comprehensive management strategies.

Various models have been proposed to explain the chronification of musculoskeletal pain conditions [5,6,7]. Among these, the “fear-avoidance model”, initially described by Lethem et al. in 1983 [8] and revised by Vlaeyen et al. in 1995 [9], stands out. This model proposes a pathway through which pain can become chronic and disabling, highlighting that the cognitive factor initiating the chronification circuit is pain catastrophizing [5]. The automatic negative thoughts triggered by pain are magnified and ruminated upon, creating a vicious cycle of persistent pain, pain anticipation, fear-avoidance of movement, hypervigilance, inactivity, and disability [5,10].

Catastrophizing is a multidimensional construct defined by an exaggerated negative mental disposition toward pain, whether real or anticipated. It comprises three components: cognitive rumination (the systematic and uncontrollable repetition of negative thoughts), magnification of automatic negative thoughts (an overestimation of pain’s threat and severity), and helplessness (a perceived inability to cope with pain), all commonly assessed using the Pain Catastrophizing Scale (PCS) [6,7,11,12,13]. As a well-established predictor of chronic pain, catastrophizing is associated with increased pain intensity, emotional distress, and poorer treatment outcomes [7]. Its role in the onset and persistence of pain is crucial, as it leads to an overreaction to pain perception, which intensifies the pain experience and fosters avoidance behaviors, in line with the fear-avoidance model [12,14].

Several strategies have been proposed to address pain catastrophizing, with cognitive interventions being particularly influential. These include pain neuroscience education, cognitive-behavioral therapy (second and third waves), and acceptance and commitment therapy [15,16]. Additional approaches encompass multimodal interventions, graded exposure, exercise, hypnosis, manual therapy, and mindfulness [17]. Despite these efforts, therapies targeting pain catastrophizing have demonstrated relatively modest effects, with more significant improvements observed when initial catastrophizing levels are high [15,17].

Recently, virtual reality (VR)-based interventions have emerged as a promising, accessible, and cost-effective alternative for managing chronic pain conditions [18]. These interventions have demonstrated encouraging results in reducing pain intensity, anxiety, kinesiophobia, and improving mood (especially with immersive VR), as well as motor control and function (notably with non-immersive VR) [19]. Despite these advancements, systematic reviews on VR for chronic pain are still limited, with outcomes varying based on intervention type, pathology, and study design, often revealing significant heterogeneity [19,20]. For patients with chronic pain, higher levels of VR immersion may improve engagement, motivation, and the sense of presence, as well as provide a distracting effect (external focus) on pain, which could lead to improved coping strategies and more effective rehabilitation [21]. Nevertheless, the analgesic mechanisms underlying VR’s effectiveness in chronic pain remain unclear [18].

Although some studies [22,23] and systematic reviews [21] have explored the impact of VR interventions on catastrophizing, this outcome has often been a secondary focus [20,21]. For instance, Amorim et al. (2025) [24] focused on pain relief, medication use, and quality of life, but neither conducted a meta-analysis nor addressed pain catastrophizing. Bilika et al. (2023) [21] performed a scoping review covering various chronic musculoskeletal pain outcomes, yet included only two studies assessing catastrophizing and provided no quantitative synthesis. Likewise, Brea-Gómez et al. limited their analysis to pain intensity outcomes in individuals with chronic neck [25] or low back pain [26], without exploring catastrophizing. Gava et al. (2022) [27] reviewed studies targeting fear, anxiety, depression, and catastrophizing, though they restricted inclusion to gamified VR interventions. Finally, Henríquez-Jurado et al. (2024) [28] analyzed outcomes such as pain intensity, kinesiophobia, disability, and health-related quality of life in spinal pain populations, but did not assess pain catastrophizing.

The high heterogeneity in study designs, intervention modalities, and reported outcomes highlights the need to systematically consolidate the existing data. Clarifying the efficacy of VR in addressing catastrophizing in chronic pain patients is crucial, particularly given VR’s potential to enhance motivation and provide distraction [18]. To date, no systematic reviews have specifically addressed or quantified the impact of VR, in all its forms, on catastrophizing in chronic pain populations. Therefore, this systematic review and meta-analysis aim to synthesize the available literature to determine the effects of VR interventions on pain catastrophizing among individuals with chronic pain.

The remainder of the study is structured as follows: The Survey Methodology section describes the literature review protocol and method that guided our work. In the Results section, we present the outcomes of our systematic review and meta-analysis. In the Discussion section, we analyze our findings, contrasting them with those of other authors. Finally, we present our work’s conclusions in the last section.

## 2. Materials and Methods

### 2.1. Protocol and Registry

This systematic review with meta-analysis was reported following the Preferred Reporting Items for Systematic Reviews and Meta-Analyses (PRISMA) statement [29]. Furthermore, the study protocol was registered in the International Platform of Registered Systematic Review and Meta-analysis Protocols platform (inplasy.com) (registration number 202480099).

### 2.2. Eligibility Criteria for the Studies

#### 2.2.1. Inclusion Criteria

The studies were selected systematically based on the PICO search tool [30], an acronym for population, intervention, comparison, outcome, and study. In that sense, we included studies if they met all the following criteria:

- Population: Adults with chronic painful musculoskeletal conditions (cause of pain is primary).
- Intervention: Interventions based on VR, both immersive and non-immersive VR. Active or passive interventions in which the participants do or do not do physical activities, respectively. The VR intervention can be applied alone or with another conventional intervention.
- Comparison: Non-intervened control, interventions without VR, standard treatment, usual care, or placebo.
- Outcome: Pain catastrophizing.
- Study design: Two-armed randomized clinical trial (RCT) with parallel groups. Also, pilot RCTs.

In addition, only peer-reviewed articles obtained from journal, conference, or workshop were considered for eligibility, with no language or temporality restriction. 

#### 2.2.2. Exclusion Criteria

Articles were excluded if they presented any of the following criteria:

- Adults with locomotor system prostheses.
- Adults with chronic pain associated with non-musculoskeletal conditions (e.g., oncologic or migraine).
- Application of VR with the sole purpose of distracting the patient during another health procedure or intervention.
- Abstracts, posters, or theses

### 2.3. Sources of Information

The electronic databases Pubmed, CINAHL, Scopus, Web of Science, and PEDro were used without filter settings from inception to 1 May 2025. In addition, a generic search was performed on Google Scholar and the reference lists of the included studies. 

### 2.4. Search Strategy

The search terms linked by Boolean operators (OR and AND) and organized according to the key elements of the question were: (i) population: Musculoskeletal Pain (MeSH); Chronic Pain (MeSH), (ii) intervention: Virtual Reality (MeSH); Video games (MeSH); Exergaming (MeSH), and (iii) outcomes: Catastrophization (MeSH); Pain Catastrophizing. The search strategy is presented in Supplementary Table S1.

### 2.5. Selection of the Studies

All records obtained from the scientific databases were imported into the Rayyan application [31]. After eliminating duplicates, records were screened by titles and abstracts to identify studies that potentially met the inclusion criteria. Articles that met the inclusion criteria were screened for eligibility by reading their full text when available. Two independent reviewers (POR and FGR) completed this process, and a third author (CCP) resolved discrepancies. Inter-rater agreement was assessed using the Kappa index interpreted according to Landis and Koch [32].

### 2.6. Data Extraction

A standardized form was used to extract information from the selected studies, including: (i) author, year, and country, (ii) characteristics of the participants and samples, (iii) types of interventions and protocols, (iv) Measuring scales, and (v) main results. Two reviewers (POR and FGR) performed data extraction independently, and a third author (CCP) intervened to standardize the information. When necessary, the corresponding authors of the selected studies were contacted for specific information.

### 2.7. Risk of Bias Assessment

The risk of bias (RoB) was assessed using the Cochrane Collaboration’s RoB-2 tool. Each of the five criteria was rated as Y = yes, PY = probably yes, PN = probably no, N = no, or NI = no information. Based on these ratings, the overall RoB for the study was determined as low risk, some concerns, or high risk [33]. Two authors (POR and FGR) independently applied the tool, and a third reviewer (CCP) resolved any discrepancies. The graphs were generated using the Robvis web application [34].

### 2.8. Data Synthesis and Analysis

Studies were meta-analyzed using Review Manager^®^ software version 5.4.1 and web-based tool MetaAnalysisOnline.com [35]. Effect size (ES) was expressed as either the mean difference (MD) or the standardized mean difference (SMD), depending on the consistency of the measurement instruments across studies. A random-effects model was applied in all cases. When applicable, medians and interquartile ranges were converted to means and standard deviations using validated methods [36,37].

Sensitivity analyses were conducted to assess the robustness of the meta-analytic findings. These included separate meta-analyses and subgroup analyses according to: (1) risk of bias of the included studies, (2) type of virtual reality system, (3) therapeutic approach of the VR intervention, (4) type of control group, and (5) measurement instrument used to assess pain catastrophizing. For consistency and valid comparability with the primary analysis, all sensitivity analyses were conducted using standardized mean differences. Publication bias was additionally assessed through visual inspection of funnel plot and Egger’s regression test. 

Heterogeneity was assessed with the inconsistency index (I^2^), categorized as might not be important (0–40%), moderate (30–60%), substantial (50–90%), and considerable (75–100%). For calculating the ES (Hedges’ g), the mean, standard deviation, and sample size post-intervention of the study groups were considered, with classifications as follows: 0.20–0.49 small; 0.50–0.79 moderate; and 0.80 high [38]. These data were essential for determining the eligibility of studies for inclusion in the meta-analyses.

## 3. Results

### 3.1. Search Results

**Figure 1:**
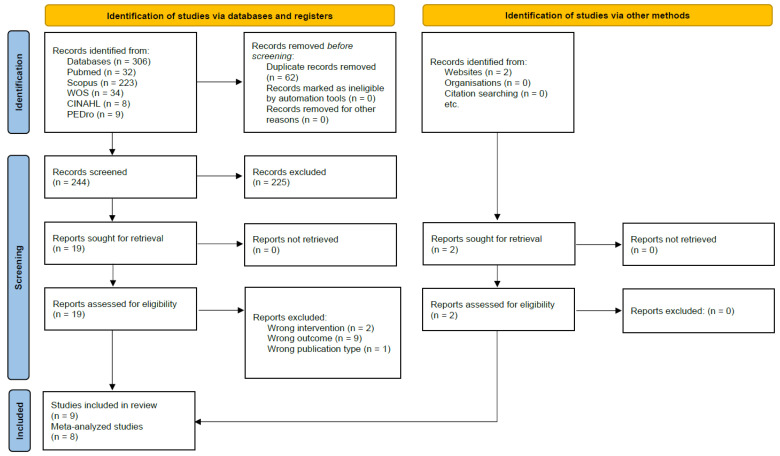
Flowchart for study selection.

A total of 306 records were identified from the databases, of which, 62 duplicates were removed. Subsequently, 244 records were screened by title and abstract, and 19 full-text articles were reviewed in detail. Seven of these met the eligibility criteria [22,23,39,40,41,42,43]. Additionally, two studies were identified and included from a generic search on Google Scholar [44,45], resulting in a final inclusion of nine studies in this review, with eight of them being meta-analyzed [22,23,39,40,42,43,44,45]. The inter-rater agreement was ‘almost perfect’ for both the screening and eligibility phases (k = 0.834 and k = 0.851, respectively). Figure 1 shows the PRISMA flow diagram of the selection process [46].

### 3.2. Characteristics of Included Studies

Of the nine articles included in this review, four were from the United States [23,40,41,45], one from Turkey [43], one from Netherlands [44], one from Spain [39], and two from Japan [22,42]. The publication interval was between 2020 and 2025, with two articles in 2020 [39,41], two in 2021 [22,40], one in 2023 [44], two in 2024 [23,45], and two in 2025 [42,43], which indicates the increasing importance of the topic of pain catastrophizing in recent years. The characteristics of these studies are presented in Table 1. Although a wide range of outcomes were explored in the included primary studies, our meta-analysis focused exclusively on pain catastrophizing. A full list of the additional outcomes reported in the included studies is provided in Supplementary Table S2, where the differences favoring the virtual reality intervention groups are also indicated.

### 3.3. Risk of Bias

**Figure 2:**
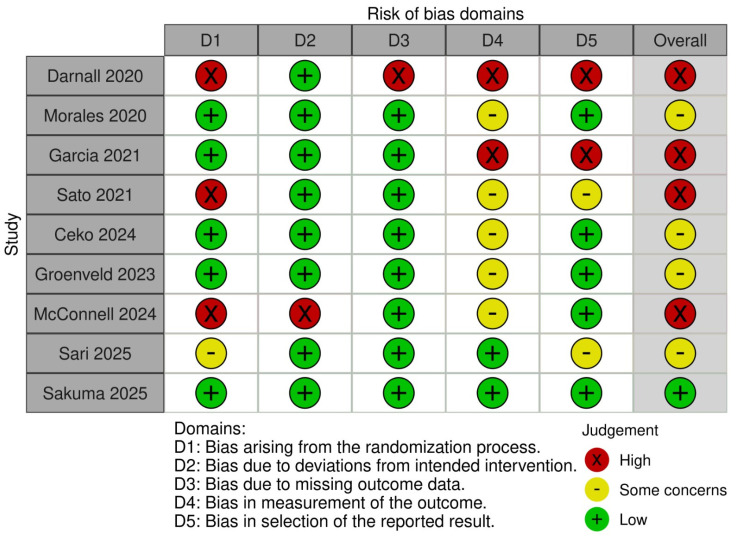
Risk of bias traffic light plot [22,23,39,40,41,42,43,44,45].

**Figure 3:**
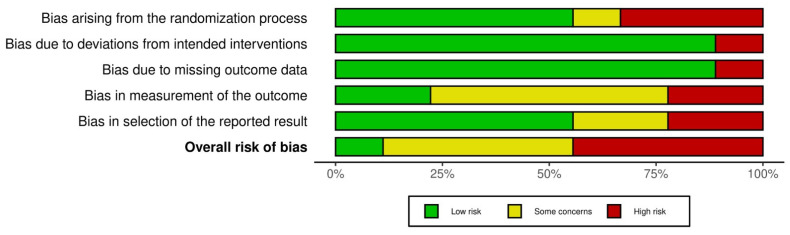
Risk of bias summary plot.

According to the detailed assessment using the RoB-2 tool, four studies demonstrated a high overall risk of bias due to issues across several key domains. The study by Darnall et al. [41] showed high risk in domains D1, D3, D4, and D5. García et al. [40], although presenting low risk in domains D1, D2, and D3, was classified as high risk in D4 and D5. Sato et al. [22] was rated as high risk in domain D1 and showed some concerns in D4 and D5. Finally, the study by McConnell et al. [45] exhibited high risk in domains D1 and D2, with some concerns in D4. Conversely, the studies by Morales et al. [39], Čeko et al. [23], Groenveld et al. [44], and Sari et al. [43] showed lower RoB, with favorable evaluations in most domains, although some concerns were noted, particularly in D4. The study of Sakuma et al. [42] was the best evaluated, with all domains rated as low risk. Overall, domains D2 and D3 were rated most favorably across the majority of studies, while domains D1 and D4 showed the highest RoB. Figure 2 provides a visual representation of the RoB for each study, and Figure 3 presents a summary assessment of each domain and the overall risk.

### 3.4. Characteristics of the Population

Among the nine selected studies, a total of 565 individuals with chronic musculoskeletal pain were included. This cohort comprised 187 males, 377 females, and one participant classified as ‘other,’ as reported by García et al. [40]. The sample sizes across studies varied, ranging from 29 participants [43] to 179 participants [40]. Participants’ ages ranged from 18 to 82 years, in accordance with the inclusion criteria of the studies. Specifically, four studies focused on adults with chronic low back pain [22,40,44,45], one study included patients with chronic back pain [23], one involved adults with chronic neck pain [39], one focused on women with fibromyalgia [43], another included patients with any form of chronic pain [42], and one study addressed adults with chronic low back pain and/or fibromyalgia [41].

### 3.5. Characteristics of Interventions and Outcome Measures

The selected interventions encompassed both immersive and non-immersive VR formats, including exergames. Among these, eight were immersive VR interventions [23,39,40,41,42,43,44,45], and only one was an exergame [22]. The VR interventions varied in type, with motor-based [22,39] and psycho-cognitive [23,40,41,42,43,44,45] approaches, the latter being more prevalent (seven studies versus two). The VR equipment used was diverse, with no single technology predominating: Vox Play [39], Pico G2 4K [40,45], Oculus Go [41,44], Oculus Quest [42,43], Samsung GearVR [23], and Nintendo Ring Fit Adventure [22]. The duration of the interventions ranged from a minimum of 3 weeks [41] to a maximum of 12 weeks [42,45], with a modal duration of 8 weeks [22,23,40]. Weekly session frequency varied significantly, from 1 session [40,42,43] to 21 sessions per week [44], and session length ranged from 2 min [40] to 40 min [22] per session. All studies employed the PCS-13 scale [22,23,39,42,43,44,45] to assess pain catastrophizing [13], except for two, which used a four-item variant of the PCS [40,41] that does not correspond to the validated four-item scale by Bot et al. (2014) [47] and has not been previously validated.

### 3.6. Effects of Interventions and Heterogeneity 

For the meta-analysis, forest plots were generated based on the results of eight studies [22,23,39,40,42,43,44,45]. We excluded the study of Darnall et al. [41] from the meta-analysis due to insufficient or incomplete data for a quantitative synthesis. The analysis considered both the overall studies and their categorization according to the type of VR, the therapeutic approach of the VR, and the control conditions.

**Figure 4:**
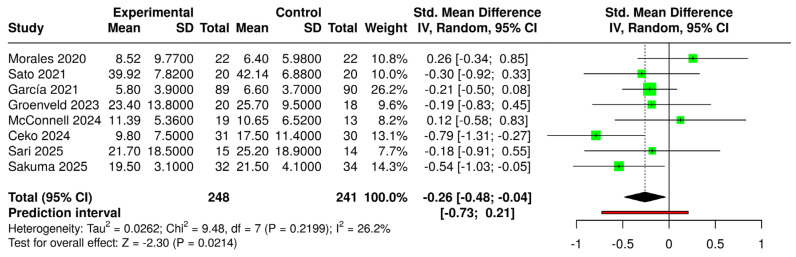
Forest plot for the comparison of all VR systems (experimental) versus controls (intervened and non-intervened) [22,23,39,40,42,43,44,45].

**Figure 5:**
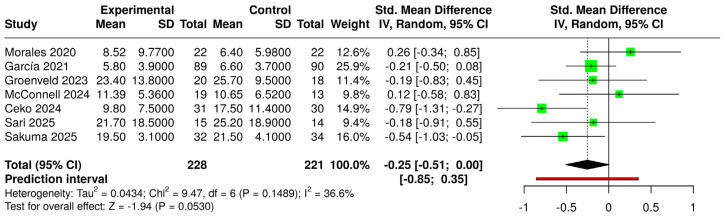
Forest plot for the comparison of immersive VR systems (experimental) versus controls (intervened and non-intervened) [23,39,40,42,43,44,45].

The comparison of all VR interventions (regardless of type and approach) with control groups (regardless of condition) yielded a significant standardized mean difference (SMD) (g = −0.26 [−0.48; −0.04]) (small effect size) (Figure 4). When considering only immersive VR compared with the same controls, the SMD (g = −0.25 [−0.51; −0.00]) was not statistically significant (Figure 5). In both forest plots, heterogeneity was not significant (*p* > 0.05) and classified as “might not be important”.

**Figure 6:**
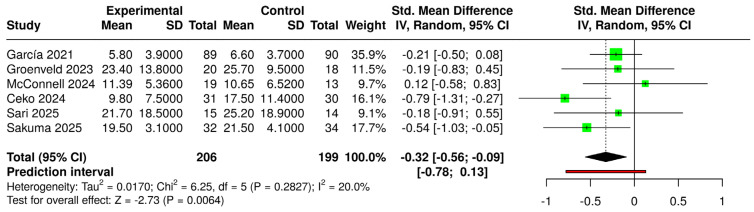
Forest plot for the comparison of psycho-cognitive VR-based interventions (experimental) versus controls (intervened and non-intervened) [23,40,42,43,44,45].

**Figure 7:**
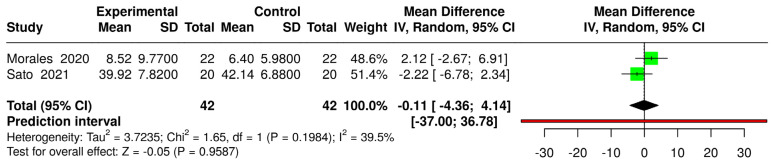
Forest plot for the comparison of physical exercise-based VR interventions (experimental) versus controls (intervened and non-intervened) [22,39].

The comparison between psycho-cognitive VR interventions and control groups (regardless of condition) resulted in a significant SMD (g = −0.32 [−0.56; −0.09]), indicating a small effect size (Figure 6). In contrast, physical exercise-based VR interventions compared with the same controls showed a non-significant mean difference (MD = −0.11 [−4.36; 4.14]) (Figure 7). In both forest plots, heterogeneity was classified as “might not be important” and was not statistically significant (*p* > 0.05).

**Figure 8:**
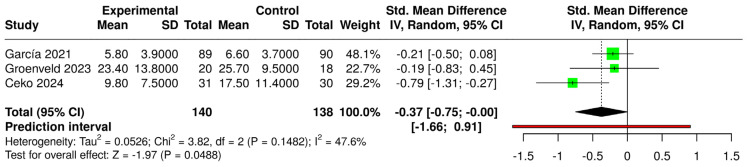
Forest plot for the comparison of psycho-cognitive interventions based on immersive VR (experimental) versus non-intervened or sham VR controls [23,40,44].

**Figure 9:**
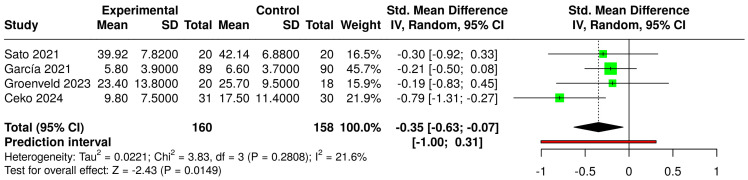
Forest plot for the comparison of all VR-based interventions (experimental) versus non-intervened or sham VR controls [22,23,40,44].

The comparison between psycho-cognitive interventions based on immersive VR and non-intervened or sham-treated control groups showed a significant SMD (g = −0.37 [−0.75; −0.00]) (small effect size) (Figure 8). Additionally, when considering all VR interventions compared to non-intervened or sham-treated controls, the SMD (g = −0.35 [−0.63; −0.07]) was also significant (small effect size) (Figure 9). In both forest plots, heterogeneity was not significant (*p* > 0.05), classified as moderate and “might not be important”, respectively.

**Figure 10:**
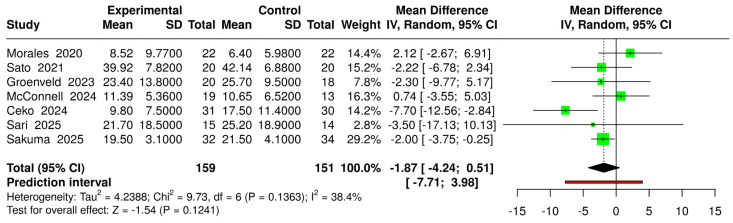
Forest plot for the comparison of all VR systems (experimental) versus controls (intervened and non-intervened), including only studies that used the 13-item Pain Catastrophizing Scale [22,23,39,42,43,44,45].

To ensure consistency in outcome measurement, an additional meta-analysis was conducted including only studies that employed the validated 13-item version of the PCS (Figure 10). This analysis revealed a non-significant MD favoring virtual reality interventions over controls (MD = −1.87 [−4.24; 0.51]; *p* > 0.05). Between-study heterogeneity was low to moderate, indicating no statistically significant variability across studies or in the overall effect (I^2^ = 38.4%, *p* > 0.05).

**Figure 11:**
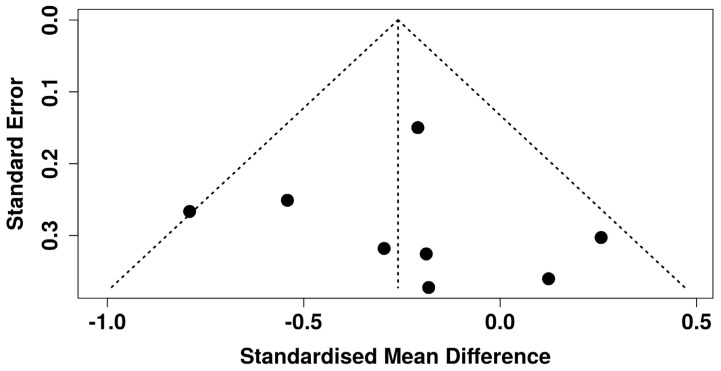
Funnel plot assessing publication bias across all studies included in the meta-analysis [22,23,39,40,42,43,44,45].

Finally, visual inspection of the funnel plot (Figure 11) did not reveal evidence of publication bias among the included studies evaluating pain catastrophizing in chronic pain populations using VR interventions. This finding was supported by Egger’s regression test, which showed no significant funnel plot asymmetry (intercept = 0.47, 95% CI: −2.29 to 3.23; t = 0.333; *p* = 0.75).

### 3.7. Sensitivity Analysis

To assess the robustness of the overall findings, a series of sensitivity analyses were performed based on different methodological and clinical characteristics. All comparisons used standardized mean differences (SMD) to allow for consistent effect size interpretation across subgroups.

The overall meta-analysis including all studies showed a small but significant effect (g = −0.26; *p* = 0.02). Excluding studies with high RoB [22,40,45] yielded a similar effect size (g = −0.32; *p* = 0.09), although statistical significance was lost. When removing the study by Sato et al. [22] to isolate interventions with immersive VR, the effect size remained comparable (g = −0.25; *p* = 0.053). Subgrouping by VR approach revealed significant effects only in cognitive-based VR interventions (g = −0.32; *p* = 0.006) [23,40,42,43,44,45], whereas motor-based interventions showed no effect (g = −0.01; *p* = 0.96) [22,39]. A separate analysis including only studies with sham or passive control groups also revealed a statistically significant effect (g = −0.35; *p* = 0.01) [22,23,40,44]. Finally, restricting the analysis to studies that used the PCS-13 for catastrophizing assessment showed a larger but non-significant effect (g = −1.87; *p* = 0.12) [22,23,39,42,43,44,45].

## 4. Discussion

The primary aim of this study was to assess the effects of VR interventions on catastrophizing in individuals with chronic pain. To achieve this, a systematic review and meta-analysis were conducted, adhering to the PRISMA guidelines for transparent reporting [29]. A total of nine randomized controlled trials (RCTs) were included, with eight subjected to meta-analysis [22,23,39,40,42,43,44,45]. One study was qualitatively analyzed due to insufficient or incomplete data for a quantitative synthesis [41]. 

The meta-analysis evaluated the effectiveness of VR-based interventions compared to various control groups. The results demonstrated a small but significant effect in the overall comparison of all VR interventions versus controls (g = −0.26), suggesting a modest yet consistent improvement in outcomes. Specifically, when immersive VR was analyzed, a comparable effect was observed (g = −0.25). Although the result did not reach statistical significance (*p* = 0.053), the consistency in effect size indicates a potential benefit that merits further exploration. Psycho-cognitive interventions utilizing VR, particularly the immersive modality, also yielded significant positive effects (g = −0.32 and g = −0.37, respectively). Additionally, the comparison of all VR interventions against non-intervened or sham-treated controls revealed a significant effect (g = −0.35), further supporting the utility of these interventions in enhancing cognitive outcomes. However, physical exercise based on VR did not show significant improvements compared to controls. The statistical heterogeneity observed in the analyses was low to moderate, indicating relative consistency in effects across studies. Although these findings are promising, the limited number of included studies suggests that the clinical and scientific community’s interest in exploring the impact of VR therapies on pain catastrophizing is still emerging. This, along with the variability in intervention protocols, pathological conditions, and therapeutic approaches, may partly explain the small effect sizes observed.

Depending on the technology, screens, control, and feedback devices used, VR systems can be classified as non-immersive, semi-immersive, and fully immersive [48]. Fully immersive systems provide a multisensory experience (visual, tactile, vestibular, and auditory) that facilitates embodiment to a greater extent than systems with lower levels of immersion [49], while non-immersive devices promote positive adherence, enjoyment, higher activity-specific balance confidence, as well as effective stimulation of motor control and functionality [19,48]. This review included all types of VR systems, encompassing both immersive and non-immersive modalities; however, only one study utilized a non-immersive VR approach, resulting in limited representation of this modality. Future research should expand the investigation of non-immersive VR to better understand its potential impact on pain catastrophizing.

VR-based interventions for chronic musculoskeletal conditions are gaining acceptance due to their positive effects on clinical outcomes such as pain reduction, increased functionality, and improved psychological factors, including kinesiophobia and catastrophizing [21,50,51,52]. VR’s mechanisms, such as distraction, multisensory stimulation, and attentional demand, help reconfigure pain perception by engaging the insular and sensory cortices, like opioid effects [53,54]. Functional improvements may arise from neurophysiological changes that alter pain signaling pathways [55,56]. Additionally, VR reduces the need for opioids and enhances psycho-cognitive outcomes, such as anxiety and depression, and quality of life [19,20,26,57,58,59,60]. While promising, the heterogeneity of existing studies emphasizes the need for rigorous trials to optimize VR’s therapeutic potential and clarify its interactions with psychological variables in chronic pain management [61,62].

Catastrophizing is a key predictor of the course of chronic pain, being associated with greater disability, depression, and pain intensity [63,64,65]. Acceptance has been shown to modulate the effects of pain-related catastrophizing, suggesting that its impact is linked to other behavior or psychological responses such as kinesiophobia or hypervigilance [64]. Longitudinal studies have found that early reductions in catastrophizing predict improvements in pain intensity and its interference with quality of life [66]. Furthermore, catastrophizing can be effectively managed through various therapeutic modalities, leading to significant reductions in both catastrophizing and pain-related outcomes [67]. 

Given that VR has emerged as a promising tool in the management of chronic pain by enhancing engagement, motivation, and providing effective distraction [52,57,68], and considering that catastrophizing is frequently reported as a secondary outcome in VR studies, synthesizing the evidence on its therapeutic use in both conditions is crucial. Therefore, in the authors’ view, this systematic review is the first to evaluate the effects of VR on catastrophizing in individuals with chronic pain.

Among the studies included in this systematic review and meta-analysis, seven [22,23,39,42,43,44,45] used the original 13-item PCS to assess catastrophizing, while two studies [40,41] employed a modified four-item version. The PCS is a widely used, self-reported questionnaire that measures the extent of pain catastrophizing through 13 statements rated on a 5-point Likert scale, reflecting how frequently individuals experience catastrophic thoughts (0 = not at all; 4 = all the time). This validated instrument is available in multiple languages, although its ability to differentiate between the subcomponents of catastrophizing (rumination, magnification, and helplessness) has been questioned [13,69,70,71]. While shorter validated and less comprehensive scales exist [72,73], a modified four-item version used in two included studies [40,41] was primarily developed to prioritize brevity and, although they share three out of four items with another previously validated instrument [47], it has not been formally validated. Consequently, the results from these two studies should be interpreted with caution. Notably, when García et al.’s study [40]—the only one reporting quantitative data using the four-item PCS—was excluded, the meta-analysis of VR interventions on pain catastrophizing showed a large but non-significant effect size (MD = −1.87), highlighting uncertainty regarding VR’s impact when limited to validated measurement tools. This finding underscores the critical need for consistent and validated outcome measures in this research area. Nonetheless, it does not discount the potential of VR therapies; rather, it emphasizes the necessity for future well-designed studies and standardized intervention protocols to better elucidate VR’s therapeutic role in reducing pain catastrophizing.

The studies analyzed in this review exhibit a heterogeneity of approaches and outcomes related to pain catastrophizing. Čeko et al. and Sakuma et al. reported significant reductions in catastrophizing through a VR-based interventions combining psycho-cognitive and physical exercise approaches, while Sato et al. and Morales et al. did not observe relevant improvements with the use of VR in an exclusively physical exercise focus [22,23,39,42]. In contrast, Darnall et al. identified a decreasing trend in catastrophizing in both the VR and audio groups, with more pronounced effects in the VR group [41]. However, Groenveld et al. and McConnell et al. found no significant differences between the VR groups and their controls [44,45]. Additionally, studies that included more frequent and prolonged VR sessions [23,41] reported sustained improvements in catastrophizing. More recently, two studies using immersive VR for relaxation purposes have shown promising results. Sari et al. [43] applied relaxation VR as a standalone intervention, whereas Sakuma et al. [42] combined relaxation VR with subsequent physical therapy, also incorporating a mixed psycho-cognitive and motor approach. Both studies reported significant improvements within intervention groups compared to baseline; however, Sakuma et al. did not find significant differences between groups. 

The meta-analysis revealed that VR-based therapy can significantly reduce catastrophizing in individuals with chronic pain compared to control groups, both treated and untreated. This finding particularly aligns with results obtained from immersive VR interventions combined with psycho-cognitive therapies—such as neuroscience-based education, cognitive-behavioral therapy, mindfulness, relaxation techniques, and self-administered behavioral skills—which were shown to be more effective in reducing catastrophizing compared to controls [23,40,43,44,45]. In contrast, VR interventions combined with physical exercise did not demonstrate significant differences from their controls [22,39,42]. Overall, while VR therapies present promising potential for reducing pain catastrophizing in individuals with chronic pain—especially immersive modalities integrated with psycho-cognitive approaches—further research is needed to validate these findings, refine intervention protocols, and better understand variations in treatment response.

The discrepancies observed—and the small and sometimes non-significant effect sizes—may be attributed to considerable variability across studies. Differences in treatment protocols, session frequency and duration, the type and level of VR immersion, and the combination with other therapeutic modalities (e.g., physical exercise vs. psycho-cognitive approaches) could have contributed to inconsistent outcomes. In addition, the use of different assessment tools, including both validated and non-validated versions of the PCS, may have introduced measurement bias. The diversity in chronic pain conditions and baseline levels of catastrophizing among participants could also have played a role in modulating treatment responsiveness. Notably, four of the nine included studies were rated as having a high risk of bias, which may have further contributed to variability in results and imprecision in effect estimates. Altogether, these factors may have influenced the observable impact of VR interventions, underscoring the need for more standardized, stratified, and population-specific approaches in future research. Future studies should also ensure improved methodological quality and better control of potential sources of bias.

Sensitivity analyses revealed a generally robust small effect of VR interventions on pain catastrophizing, with estimates consistently favoring VR across most models. Heterogeneity remained low to moderate, indicating a fair degree of consistency among included studies. Notably, cognitive-oriented VR interventions produced the most reliable and statistically significant effects, suggesting that cognitive engagement may serve as a key therapeutic mechanism. The attenuation of effects upon exclusion of high-risk-of-bias studies underscores the influence of methodological quality on estimate precision. Similarly, larger effects observed in comparisons against sham or passive controls highlight the importance of comparator choice in accurately capturing intervention efficacy. While subgroup analyses addressed some sources of variability, methodological heterogeneity across studies may have introduced residual confounding, limiting the accuracy of pooled estimates. This limitation should be carefully considered when generalizing results to broader clinical contexts.

Finally, limitations of this systematic review are as follows: (i) the exclusion of non-randomized or single-group study designs. Although the exclusive inclusion of RCTs aims to synthesize the best available evidence, this restriction also limits the diversity of data, reducing the opportunity to analyze a wider range of potential effects of interventions in diverse clinical contexts. (ii) Although statistical heterogeneity among the studies was low, the observed effect sizes of the interventions compared to control conditions were small. This suggests that while the results are consistent and statistically significant, the magnitude of the effects is modest. Therefore, although virtual reality interventions demonstrate a measurable impact on pain catastrophizing, the clinical relevance of these effects should be interpreted with appropriate caution and considered within the broader context of complementary therapeutic approaches. (iii) Of the seven studies included in this review, only one considered an active intervention based on physical exercise guided by a non-immersive video game system, preventing direct comparisons. Since immersive and non-immersive systems offer different levels of stimulus, sensory experience, and presence, it is likely that these factors may influence the magnitude of the therapeutic effects observed. (iv) Overall, the high RoB in the included studies suggests that their results may have been influenced by various methodological factors, which could have led to an overestimation or underestimation of the actual effects of the interventions. Consequently, these findings should be interpreted with caution.

## 5. Conclusions

Although current evidence is limited in confirming that VR-based interventions are clinically more effective than other therapeutic modalities in reducing pain catastrophizing in people with chronic pain, this systematic review and meta-analysis shows promising results, particularly in the use of immersive VR with a psycho-cognitive approach. However, due to the small number of studies, their high RoB, and variability in intervention protocols, these findings should be interpreted with caution. Future RCTs should address the limitations of this review to further explore the effects of VR on pain catastrophizing.

## Figures and Tables

**Table 1:** Characteristics of the included studies.

Author	Clinical Characteristics	Age (Years), Mean (SD)	Sample (n); M/F/O	Type of Intervention	Duration; Frequency; Exercise Session Time/Repetitions	Scale	Intragroup Result (Pre-Test vs. Post-Test)	Intergroup Post-Test Result
Darnall et al., 2020 [41]	Males and females, aged 18–75 years, self-reported chronic nonmalignant low back pain or fibromyalgia, with an average pain intensity >4 over the past month and chronic pain duration >6 months.	G1: NR G2: NR	G1: n = 35; 26/9 G2: n = 39; 26/13	G1: VR intervention group. Received 4–8 treatment sessions from each content category (CBT, Relaxation, Mindfulness) using a VR system (Oculus Go). G2: Audio group. Received 4–8 treatment sessions from each content category (CBT, Relaxation, Mindfulness) via audio.	G1: 21 days, 1 daily session, 1–15 min/session G2: 21 days, 1 daily session, 1–15 min/session	4-item PCS (adapted)	Significant main effect of time for both groups (*p* < 0.001) (ANOVA). G1: Significant improvement (*p* < 0.001) G2: Significant improvement (*p* < 0.001)	No significant group effect (*p* = 0.61) (ANOVA). G1 did not differ from G2 (*p* = 0.61).
Morales et al., 2020 [39]	Males and females, aged 18–65 years, chronic non-specific neck pain (criteria NR)	G1: 32.72 (11.63) G2: 26.68 (9.21)	G1: n = 22; 11/11 G2: n = 22; 10/12	G1: VR treatment group. Cervical exercises (flexion-extension, lateral bending, axial rotation) performed using “Fulldive VR” and “VR Ocean Aquarium 3D” (VR Vox Play system and LGQ6 smartphone). G2: Neck Exercises group. Conventional cervical exercises (flexion-extension, lateral bending, axial rotation).	G1: 4 weeks; 2 sessions/week; 3 sets/10 reps/exercise G2: 4 weeks; 2 sessions/week; 3 sets/10 reps/exercise	PCS-13	G1: Significant improvement (*p* < 0.05) (d = 0.77) G2: Significant improvement (*p* < 0.05) (d = 0.7)	G1 did not differ from G2 (*p* > 0.05)
García et al., 2021 [40]	Males and females aged 18–85 with self-reported CLBP without radicular symptoms, pain duration ≥6 months, and pain intensity ≥4 on the DVPRS scale	G1: 51.5 (13.5) G2: 51.4 (12.9)	G1: n = 89; 22/67 G2: n = 90; 19/70/1	G1: Therapeutic VR. CBT, mindfulness, PNE, BPS education, breathing-relaxation exercises, executive function exercises (EaseVRx/Applied VR). G2: Sham VR. Non-immersive, non-interactive 2D VR with 20 nature scenes and neutral music.	G1: 56 days; 1 daily session; 2–16 min/session G2: 56 days; 1 daily session; 2–16 min/session	4-item PCS (adapted)	G1: No significant improvement (*p* > 0.05); G2: No significant improvement (*p* > 0.05)	G1 did not differ from G2 (*p* > 0.05)
Sato et al., 2021 [22]	Males and females with CLBP (≥3 months), referred to the hospital without response to previous conservative treatment	G1: 49.31 (12.59) G2: 55.61 (10.96)	G1: n = 20; 9/11 G2: n = 20; 12/8	G1: Ring Fit Adventure (RFA) group. Performed dynamic physical exercises using RFA games on a non-immersive VR system (NIVR, Nintendo Switch) while maintaining prescribed medication. G2: Control group. Continued prescribed medication plus NSAIDs, Tramadol, and Duloxetine (dose controlled bi-weekly based on interview results).	G1: 8 weeks; 1 session/week; 40 min/session G2: 8 weeks; medication + NSAIDs, Tramadol, Duloxetine	PCS-13	G1: No significant improvement (*p* > 0.05); G2: No significant improvement (*p* > 0.05)	G1 did not differ from G2 (*p* > 0.05)
Groenveld et al., 2023 [44]	Males and females aged 18+ years with non-specific CLBP of intensity ≥4 on an 11-point Likert scale, without radicular pain worse than CLBP, and no treatment other than analgesics or physical therapy	G1: 51.0 (2.9) G2: 52.0 (2.5)	G1: n = 20; 3/17 G2: n = 20; 4/16	G1: Cognitive-behavioral therapy via the Reducept VR app (Oculus Go). Therapy included 5 psychological treatment games (acceptance and commitment, mindfulness, hypnotherapy, eye movement desensitization and reprocessing) with educational content on maladaptive CNS changes. G2: Control group. Waitlisted for advanced pain treatment, no intervention.	G1: 4 weeks; up to 3 daily sessions; at least 10 min/day, maximum 30 min/session G2: NA	PCS-13	NR	G1 did not differ from G2 (*p* > 0.05)
Čeko et al., 2024 [23]	Males and females aged 21–70 years, CLBP (present for at least 50% of the past 6 months) with an average pain intensity ≥4/10	G1: 34.8 (9.9) G2: 33.5 (9.2)	G1: n = 31; 16/15 G2: n = 30; 15/15	G1: Neuroscience-based VR therapy group (VRNT). Received pain neuroscience education plus cognitive, behavioral, and affective exercises via an immersive VR system (Samsung Gear VR + Samsung Galaxy S-9 phone). No physical exercise included. G2: Control group. Waitlisted or usual care.	G1: 8 weeks; 10 weekly sessions (2/day, 5 days); 20 min/session (7–27 min) G2: NA	PCS-13	Intragroup result not specified, likely ANOVA	“Compared to the Control condition, the VRNT group showed significant improvements in pain catastrophizing at post-treatment (condition by time interaction controlled for age and sex: g = 0.86, *p* = 0.002)”
McConnell et al., 2024 [45]	Males and females aged 18–75 years with low back pain lasting ≥12 weeks, no relevant comorbidities, no spine surgery in the past 12 months	G1: 48.2 (12.7) G2: 43.3 (17.4)	G1: n = 19; 8/11 G2: n = 13; 4/9	G1: VR pain neuroscience education (VR-PNE) group. Received standard PT plus VR-based pain neuroscience education via the “PNE 2.0” software administered through PICO G2 4K. Therapy included education, patient testimonials, emotional regulation exercises, breathing, and meditation. G2: PT group. Standard PT only.	G1: 6 weeks; variable frequency; 21 min/session on average G2: 6 weeks; frequency and session duration as needed	PCS-13	NR	G1 did not differ from G2 (*p* > 0.05)
Sari et al., 2025 [43]	Females diagnosed with fibromyalgia for at least 1 year, aged 18–65 years, with stable health status over the previous 6 months. Patients with physical, neurological, or psychiatric conditions interfering with treatment were excluded.	G1: 38.8 (5.63) G2: 39.21 (8.42)	G1: n = 15 G2: n = 14	G1: Exposure to a relaxing virtual environment using Oculus Quest 2. The session included video with relaxing sounds simulating a walk through an animated natural forest. G2: Progressive muscle relaxation and breathing exercises without VR.	G1: 4 weeks; 1 session per week; 30 min per session G2: 4 weeks; 1 session per week; 30 min per session	PCS-13	G1: Significant improvement (*p* < 0.05, d = 0.59). G2: No significant improvement (*p* > 0.05, d = 0.04)	Significant difference in favor of G1 (*p* = 0.002, d = 1.271)
Sakuma et al., 2025 [42]	Males and females aged 18–75 years, employed, with chronic pain (≥3 months) and average pain intensity ≥ 4/10. Exclusion criteria included acute pain, cognitive impairment, or physical incompatibility with VR.	G1: 52.6 (10.9) G2: 56.8 (14.1)	G1: n = 32; 18/14 G2: n = 34; 14/20	G1: Simulated forest walk using Oculus Quest 2 followed by a supervised exercise program (stretching, strengthening, aerobic activity). Load was progressive and individualized. Home-based exercises were also prescribed. G2: Same exercise protocol without VR exposure.	G1: 12 weeks; 1 weekly VR session (10 min) + 1–2 weekly supervised exercise sessions (40 min/session: 20 min of strengthening/flexibility and 20 min of treadmill or ergometer). G2: 1–2 weekly supervised exercise sessions (40 min/session: 20 min of strengthening/flexibility and 20 min of treadmill or ergometer).	PCS-13	G1: Significant improvement (*p* < 0.001, d = 1.13). G2: No significant improvement (*p* > 0.05, d = 0.04)	G1 did not differ from G2 (*p* > 0.05)

G: Group; NR: Not reported; NA: Not applicable; M: Male; F: Female; O: Other; SD: Standard deviation; VR: Virtual reality; CBT: Cognitive behavioral therapy; BPS: biopsychosocial education; PNE: pain neuroscience education; CNS: Central nervous system; PT: Physical therapy; PCS: Pain Catastrophizing Scale; d: Cohen’s d; g: Hedges’ g; NSAIDs: Non-steroidal anti-inflammatory drugs; CLBP: Chronic low back pain.

## Data Availability

Not applicable.

## Supplementary Materials

The following supporting information can be downloaded at: https://www.mdpi.com/article/10.3390/jcm14113782/s1, Table S1: Detail of the search strategy of the electronic databases; Table S2: Other outcomes from the chosen primary studies.

## Abbreviations

The following abbreviations are used in this manuscript:

IASP	International Association for the Study of Pain
PCS	Pain catastrophizing scale
VR	Virtual reality
RCT	Randomized clinical trial
RCTs	Randomized controlled trials
RoB	Risk of bias
ES	Effect size
SMD	Standardized mean difference
CBT	Cognitive behavioral therapy
CLBP	Chronic low back pain
PNE	Pain neuroscience education
