# Supplementary material for: Effects of Virtual Reality-Based Interventions on Pain Catastrophizing in People with Chronic Pain: A Systematic Review and Meta-Analysis
Source: J Clin Med. 2025 May 28;14(11):3782. doi: 10.3390/jcm14113782 (PMC12155961; doi:10.3390/jcm14113782)
Supplement: Supplementary file 1 [file jcm-14-03782-s001.zip › Supplementary Table S1.pdf]

Supplementary Table S1.

Detail of the search strategy of the electronic databases.

**Item**	**Strategy / Code**	**Pubmed**
Population	Search: (Musculoskeletal Pain) OR (Chronic Pain)	200.671
Intervention	Search: ((Virtual Reality) OR (Video games)) OR (Exergaming)	36.282
Outcome	Search: (Catastrophization) OR (Pain Catastrophizing)	6.440
	Search: ((#5) AND (#6)) AND (#7)	32
**Item**	**Strategy / Code**	**Scopus**
Population	ALL (musculoskeletal pain OR chronic AND pain)	1.214.662
Intervention	ALL (virtual AND reality OR video AND games OR exergaming)	165.875
Outcome	ALL (catastrophization OR pain AND catastrophizing)	28.199
	(ALL (musculoskeletal pain OR chronic AND pain)) AND (AL L (virtual AND reality OR video AND games OR exergaming)) A ND (ALL (catastrophization OR pain AND catastrophizing))	223
**Item**	**Strategy / Code**	**WOS**
Population	ALL=(Musculoskeletal Pain OR Chronic Pain)	206.193
Intervention	ALL=(Virtual Reality OR Video games OR Exergaming)	84.802
Outcome	ALL=(Catastrophization OR Pain Catastrophizing)	5.831
	#3 AND #2 AND #1	34
**Item**	**Strategy / Code**	**CINAHL**
Population	Musculoskeletal Pain OR Chronic Pain	57.815
Intervention	Virtual Reality OR Video games OR Exergaming	19.733
Outcome	Catastrophization OR Pain Catastrophizing	2.791
	Total	8
**Item**	**Strategy / Code**	**PEDro**
	Musculoskeletal pain AND Virtual Reality AND Pain Catastrophizing	8
	Musculoskeletal pain AND Exergam* AND Pain Catastrophizing	1
		9
	TOTAL SEARCH	306
