# Supplementary material for: Effects of Virtual Reality-Based Interventions on Pain Catastrophizing in People with Chronic Pain: A Systematic Review and Meta-Analysis
Source: J Clin Med. 2025 May 28;14(11):3782. doi: 10.3390/jcm14113782 (PMC12155961; doi:10.3390/jcm14113782)
Supplement: Supplementary file 1 [file jcm-14-03782-s001.zip › Supplementary Table S2.pdf]

Supplementary Table S2.

Other outcomes from the chosen primary studies. Asterisk (*) indicates a significant difference in favor of the virtual reality intervention group.

Included study	Other outcomes
Darnall et al. 2020 [41]	- Average Pain Intensity*<br>- Pain Interference on Activity*, Mood*, Sleep*, and Stress*<br>- Pain Self-Efficacy<br>- Patient Global Impression of Change
Morales et al. 2020 [39]	Primary outcomes<br>- Visual Analog Scale (VAS)<br>- Conditioned Pain Modulation<br>- Temporal Summation*<br><br>Secondary outcomes<br>- Active Cervical Range of Movement<br>- Neck Disability Index (NDI)<br>- Kinesiophobia*<br>- Fear-Avoidance Beliefs<br>- Pain Pressure Thresholds<br>- Pain-Related Anxiety
García et al. 2021 [40]	Primary outcomes<br>- Pain duration<br>- Pain intensity*<br>- Pain interference with activity*, mood*, sleep, stress*<br><br>Secondary outcomes<br>- Patient's Global Impression of Change<br>- Physical function*<br>- Sleep disturbance*<br>- Pain self-efficacy<br>- Chronic pain acceptance<br>- Pain medication use*<br>- Treatment satisfaction*
Sato et al. 2021 [22]	Primary outcomes<br>- Low back pain (VAS)*<br><br>Secondary outcomes<br>- Buttock pain*<br>- Leg numbness<br>- Body composition<br>- Muscle strength<br>- Grip strength<br>- Pain self-efficacy*<br>- Kinesiophobia
Groenveld et al. 2023 [44]	Primary outcomes<br>- Quality of life at 4 weeks<br><br>Secondary outcomes<br>- Daily pain (VAS)

	- Analgesics use<br>- Feasibility of the VR technology
Čeko et al. 2024 [23]	Primary outcomes<br>- Pain intensity (BPI-SF)*<br>- Pain interference (BPI-SF)*<br><br>Secondary outcomes<br>- Pain bothersomeness (Bother)<br>- Quality of life (SF-12)*<br>- Disability (OLBPQ)*<br>- Anxiety (PROMIS)<br>- Depression (PROMIS)<br>- Anger (PROMIS)<br>- Sleep problems (PROMIS)*<br>- Fatigue (PROMIS)*
McConnell et al. 2024 [45]	- Oswestry Disability Index<br>- Numeric Pain Rating Scale (NPRS)<br>- Pain Self-Efficacy Questionnaire (PSEQ)<br>- Global Rating of Change (GROC)<br>- Neurophysiology of Pain Questionnaire (NPQ)<br>- Fear Avoidance Beliefs Questionnaire (FABQ)<br>- Brief Resilience Scale (BRS)<br>- Back Beliefs Questionnaire (BBQ)
Sari et al. 2025 [43]	- Fibromyalgia Impact Questionnaire (FIQ)*<br>- Central Sensitization Inventory Short Form (CSI-SF)*<br>- TAMPA Scale<br>- Body Awareness Questionnaire (BAQ)*<br>- Visual Analog Scale (VAS)<br>- Galvanic Skin Response (GSR)<br>- Simulator Sickness Questionnaire (SSQ)
Sakuma et al. 2025 [42]	Primary outcomes<br>- Tampa Scale for Kinesiophobia (TSK)*<br><br>Secondary outcomes<br>- World Health Organization Health and Work Performance Questionnaire (WHO-HPQ)*<br>- Pain Self-Efficacy Questionnaire (PSEQ)*<br>- Numerical Rating Scale (NRS)<br>- Hospital Anxiety and Depression Scale (HADS)*<br>- International Physical Activity Questionnaire (IPAQ)<br>- EuroQOL 5 Dimensions 5-Level (EQ-5D-5L)<br>- quadriceps muscle strength<br>- Timed Up and Go test (TUG)<br>- 30-s Chair Stand test (CS-30)*
